# Using the Framework Method for the Analysis of Qualitative Dyadic Data in Health Research

**DOI:** 10.1177/10497323211011599

**Published:** 2021-05-13

**Authors:** Nicole Collaço, Richard Wagland, Obrey Alexis, Anna Gavin, Adam Glaser, Eila K. Watson

**Affiliations:** 1 School of Health Sciences, U﻿niversity of Southampton, Southampton, United Kingdom; 2 Faculty of Health and Life Sciences, Oxford Brookes University, Oxford, United Kingdom; 3 Northern Ireland Cancer Registry School of Medicine, Dentistry and Biomedical Sciences, Centre for Public Health, Queen’s University Belfast, Belfast, United Kingdom; 4 Leeds Institute of Cancer and Pathology, Faculty of Medicine and Health, University of Leeds, Leeds, United Kingdom

**Keywords:** qualitative, cancer, experiences, illness and disease, health, dyad, couples, framework method, dyadic analysis, qualitative analysis, England

## Abstract

There are an increasing number of qualitative studies which focus on the dyad (couples, families, caregivers–patients, health care professionals–patients). However, there is limited literature regarding qualitative methodology for dyadic analysis when members of the couple have been interviewed separately. The aim of this article is to share the knowledge we gained from undertaking a novel approach to dyadic analysis. We used an adapted version of the Framework method on data gathered in a study exploring the impact of prostate cancer on younger men and their partners. In this article, we examine and reflect on the challenges of this type of analysis and describe how we analyzed the interview data from a dyadic point of view, to share what we learned in the process.

## Background

An emerging body of literature exists on studies that focus on dyads, or two or more people/elements in the context of health care. Examples include couples affected by cancer (Kim et al., 2008; Manne et al., 2010; Regan et al., 2014), health care professionals and patients (Christopoulos et al., 2015), and caregiver–patient relationships (Liljeroos et al., 2014). However, good quality information about how to conduct dyadic analysis in qualitative research when members of the dyad are interviewed separately is sparse (Eisikovits & Koren, 2010). Much of the detail in the few published studies is focused on data collection (Allan, 1980; Manning & Kunkel, 2015; Morris, 2001), with little discussion about the process, relevance, and usefulness of conducting a dyadic analysis. Furthermore, few studies have discussed the importance of developing an in-depth understanding of the use of dyads as the unit of analysis (Eisikovits & Koren, 2010; Ummel & Achille, 2016) and conceptualizing the entire study from a dyadic perspective (Eisikovits & Koren, 2010).

Whether individuals within a dyad are interviewed together (dyadic data collection) or separately (non-dyadic data collection) will influence the way in which the data are analyzed. There are advantages and disadvantages to both approaches (Eisikovits & Koren, 2010; Manning & Kunkel, 2015; Ummel & Achille, 2016); however, the choice ultimately depends on the research topic being explored. Eisikovits and Koren (2010) propose that analyzing the couple data as a unit using separate interviews (non-dyadic) can both enrich and limit the perception of the study under focus, compared with analyzing the individual as a unit. Analyzing interviews at the individual level limits the perception of their experience as a couple, as the data gained are restricted to what one partner said, and their version cannot be qualified or disregarded by the other partner. Synthesizing these two accounts using dyadic analysis provides enrichment through the additional perspectives of the dyad from the researchers’ interpretations and without restricting the dyadic perspective. In some qualitative studies on dyads, individuals constitute the unit of dyadic analysis, by interviewing one person in the dyad to give their account on their experience as a whole from a dyadic point of view. The limitation of this approach is that a one-sided perspective is provided on topics that involve two parts of their whole experience. Few studies appear to connect specific methods of non-dyadic data collection and dyadic conceptualization (Eisikovits & Koren, 2010).

As part of a study which sought to understand the experiences and needs of younger men affected by prostate cancer (PCa) and their partners (using non-dyadic interviews; Collaço et al., 2020), we conducted dyadic analysis using the Framework method (Gale et al., 2013; Ritchie et al., 2003; Ritchie & Spencer, 1994). The Framework method is an approach to managing and analyzing qualitative data through a process of summarization, resulting in a series of themed matrices which allows data to be analyzed by case and theme.

A review of qualitative literature identified six articles which provided details on the analysis of non-dyadic interview data (when members of the dyad had been interviewed separately) using the Framework method (Conroy et al., 2020; Patel & Agbenyega, 2013; Primeau et al., 2017; Starmann et al., 2017; Swallow et al., 2011; White & Newman, 2016). However, little description was provided regarding the process of conducting the dyadic aspect of the analysis, making replication difficult. This led us to reflect on the methodological challenges of the analysis process when members of the couple have been interviewed separately.

This article shares our reflections on the process and challenges of conducting dyadic analysis using Framework method, to inform other researchers and encourage further development and use of this type of analysis. The reflections are discussed and exemplified using the context from our study on younger men affected by PCa and their partners (Collaço et al., 2020). Our process and reflections will be presented in the following way: (a) context of application, (b) procedure for dyadic analysis, (c) adapting the dyadic analysis process, and (d) reflections on the dyadic analysis process. It is not within the scope of this article to discuss study design in depth, further details of which are reported elsewhere (Collaço et al., 2019, 2020).

## Context of Application

### The Experiences and Needs of Couples Affected by PCa Aged 65 and Under: A Qualitative Study

In this qualitative study, semi-structured telephone interviews were conducted with men with PCa and their partners (28 couples, 56 participants) separately, by the same interviewer. Telephone interviews were chosen as the data collection method for pragmatic reasons, as participants were recruited from across the United Kingdom. Interviews were audio recorded and transcribed verbatim. Participants provided informed verbal and written consent.

PCa is often considered to be an illness of older men, but the prevalence among younger men (≤65 years) being diagnosed is rising (Salinas et al., 2014). Younger men with PCa exhibit greater unmet psychological needs than the general population of men with PCa (Britain Thinks, 2014; Chambers et al., 2015). The impact of a PCa diagnosis and side effects of treatment (e.g., incontinence, erectile dysfunction, hot flushes) can pose challenges to the lives of both men with PCa and their intimate partners (Harden et al., 2006). Therefore, the main research question for our study was as follows:

**Research Question 1:** How does PCa affect the lives of younger men (≤65 years) and their partners on an individual and dyadic level?

We asked participants about the impact of PCa on their relationships, family life, social relationships, work and finances, treatment, and health care experiences (Collaço et al., 2020). The study was approved by the National Research Ethics Service (North East-Newcastle & North Tyneside 1. REC Reference Number: 15/NE/0036).

A qualitative metasynthesis synthesized 29 articles on couples affected by PCa (excluding *n* = 12 articles focused on partners’ experiences of supporting someone with PCa; Collaço et al., 2018). A variety of data collection methods were used across the studies included in this review (focus groups, couples interviewed together, separately, or both), although most interviewed members of the couple separately (*n* = 14; Albaugh et al., 2017; Boehmer & Babayan, 2004; Fergus et al., 2002; Gilbert et al., 2013; Gray et al., 2000, 2002; O’Callaghan et al., 2014; Oliffe et al., 2015; Phillips et al., 2000; Primeau et al., 2017; Rivers et al., 2011, 2012; Ussher et al., 2013; Wittmann et al., 2014). While six studies used a thematic approach, having conducted interviews separately with members of the couple (Feltwell & Rees, 2004; Gilbert et al., 2013; Gray et al., 2002; Kelly et al., 2015; Phillips et al., 2000; Ussher et al., 2013); the process was not sufficiently detailed to explain how data were analyzed at the level of the couple and could not therefore, be replicated.

## Procedure of Dyadic Analysis

Due to the absence of specific, step-by-step methodological guidance within the literature for dyadic analysis when members of the couple had been interviewed separately, we adapted the Framework method (Gale et al., 2013; Ritchie et al., 2003) to incorporate the method of two other studies (Eisikovits & Koren, 2010; Yosha et al., 2011).

### The Framework Method

**Figure 1.**
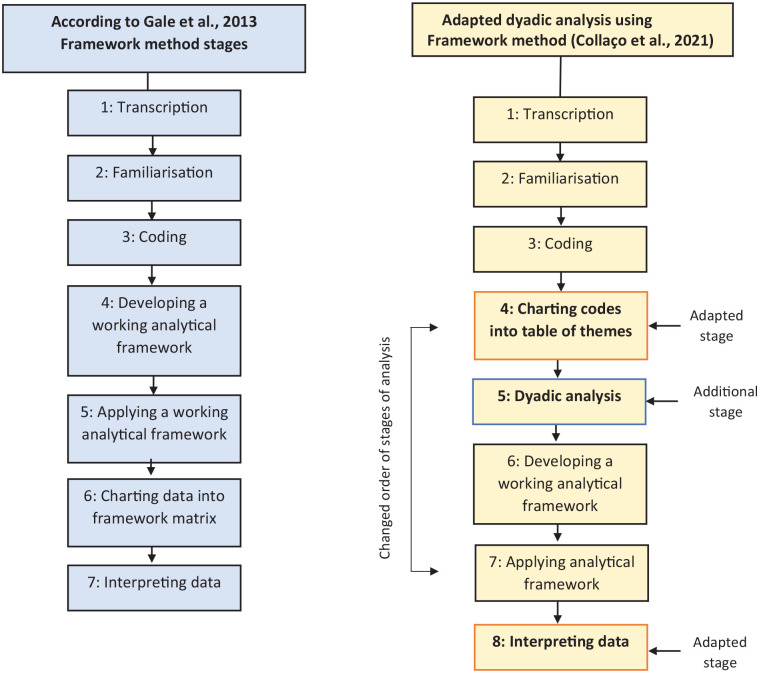
Adapting the dyadic analysis process using the Framework method.

The Framework method was developed by social researchers in the United Kingdom as an approach to analyze qualitative data applied to policy research (Ritchie et al., 2003; Ritchie & Spencer, 1994). The Framework method consists of several stages (Gale et al., 2013; Ritchie et al., 2003; see Figure 1) and is based on a common set of principles which comprise qualitative analysis: transcribing interviews, immersion in data, developing a data coding system, and linking codes to generate overarching categories/themes which may lead to theory development (Morse & Richards, 2012). A core feature which differentiates Framework method from other qualitative methodologies is the matrix development: rows (interviewee), columns (codes), and cells of summarized data, which provide a structure that enables the researcher to systematically develop and reduce the data to analyze it by case and code. This allows for greater transparency of the data analysis process and illustrates the advantage of participants’ views remaining connected to other aspects of their account within the themed matrix so that the context of the individual’s views is not lost. It also allows for comparisons and differences to be identified more clearly. Researchers can move more fluidly and flexibly back and forth across the data until a coherent narrative emerges (Gale et al., 2013; Ritchie et al., 2003).

Analysis of the data began when interviews for both members of the dyad were conducted. After analyzing each participant’s data on an individual level, we mapped the data visually through the Framework matrix (a spreadsheet which contains summarized data of codes (columns) and cases (rows)—see Supplementary File) to get a clearer understanding of the parallel progression between the partners’ experiences. In doing so, we identified overlaps and contrasts (the extent of similarity and difference between the individuals’ understanding of their experiences) within the data, which provided us with a comprehensive perspective that was more than the sum of the two individual versions (Eisikovits & Koren, 2010).

Dyadic analysis was used as the core stage of analysis. Dyadic analysis in qualitative research allows for researchers to better understand and identify overlaps and contrasts between members of the couple interviewed, particularly if they have been interviewed separately. This, in turn, enables researchers to see beyond the individual perspectives and into the perceptions of their experiences as a dyad (Manning & Kunkel, 2015).

## Adapting the Dyadic Data Analysis Process

To develop the dyadic analysis process using the Framework method, research from Eisikovits and Koren’s (2010) method of dyadic analysis and Yosha et al.’s (2011) methodology was drawn upon and adapted. Eisikovits and Koren’s (2010) article on *approaches to and outcomes of dyadic analysis* discussed the details of dyadic analysis in the context of phenomenological traditions, through analyzing the overlaps and variations between the two versions of the dyad in the study: husband/wife or partner/partner. The authors proposed that combining the two perspectives of members of the dyad can aid in the development of a dyadic perspective which addresses how the experience of each partner is established and vice versa (Schutz, 1972).

Yosha et al.’s (2011) study explored the methodology “multi-perspective analysis” in the context of cancer patients and their navigators’ process of patient navigation. This methodology was drawn upon to guide the initial phases of dyadic analysis for this study on younger men affected by PCa and their partners. Multi-perspective analysis is an infrequently used qualitative methodology that may be used to provide a deeper understanding of the needs and experiences of two or more people. We felt this methodology to be more appropriate as it can be used to offer insight and understanding of relationships and dynamics, and variance in perceptions of experiences.

Yosha et al.’s (2011) analysis process involved reading the transcripts of the separately conducted interviews of a dyad to create a dyadic summary that comprised free text compilations of emerging themes related to the research questions with supporting quotes. Reading separate transcripts for the man with PCa and partner as a dyad was a more complex process than initially perceived, as there are many different elements and processes that comprise a couple’s experience of cancer. These would be difficult to reflect in one summary, and thereafter it would be difficult to create a clear audit trail to reflect the development of themes. Yosha et al.’s (2011) analysis involved the creation of a table of patient and partner quotes to develop themes. This was adapted for the Framework method process by creating tables consisting of themes and subthemes relevant to a man with PCa and his partner/wife (see evolution of framework development below—see Supplementary File), and was a pivotal stage in bringing together couples’ experiences in a transparent and clear way. Creating one overall dyadic summary as carried out by Yosha et al. (2011) seemed simplistic and lacked the detail and context relevant to the contrasts and overlaps in experience which compromise a dyadic experience. Therefore, the seven stages of the Framework method as reported by Gale et al. (2013) were adapted through implementing an additional stage (Stage 5–dyadic analysis), changing the order of the Framework method stages and incorporating Yosha et al.’s (2011) dyadic summaries, and Eisikovits and Koren’s (2010) approach to identify overlaps and contrasts to carry out the analysis in this study (see Figure 1).

The steps are as follows (see Table 1).

**Table 1.** Stages of Dyadic Analysis.

Stage 1: Transcription	Audio files were transcribed from participant interviews verbatim. Large margins were created on transcripts to provide adequate space to code.
Stage 2: Familiarization with the interview	Familiarization with the interviews occurred through re-reading transcripts and/or looking back through reflective notes.
Stage 3: Coding	After reading the transcript, codes were applied to appropriate lines based on the experiences and needs of the couple. For example, codes reflecting improvement suggestions, the impact to the couple’s relationship, social and work implications. Each transcript was coded for the man with PCa and his partner separately.
Stage 4: Charting codes into the table of themes	A table of general themes were created based on the questions asked and the codes from the participants’ transcripts. For example, themes were created based on the questions asked around the impact on social life, financial impact, impact on their relationship, and experience of health care services. Subthemes based on codes from the transcripts of the man with PCa and his partner were placed under each theme with data summarized and quotes added. Subthemes that did not reflect the general themes were developed under a different theme. For example, codes reflecting an *uncertain future* may not fit under general themes, and therefore a new theme such as *Disrupted lives* could be created. Codes in which there was uncertainty of their placement were put under the theme *Other* until such point that a theme was derived, or clarity sought to its appropriate coding.
Stage 5: Dyadic analysis	Dyadic codes/summaries were created based on the themes and subthemes for the individual couple. This involved exploring the extent of agreement between members of the dyad, and how each theme affected one another and possibly changed the experience depending on how each couple addressed a particular problem. Further codes were developed from the dyadic analysis which reflected the couples’ experiences and needs rather than individual experiences.
Stage 6: Developing a working analytical framework for dyads	New themes were created based on the dyadic subcodes created. The defined dyadic analysis codes and possible new themes which reflect the different matrices were discussed with co-authors. This formed the working analytical framework.
Stage 7: Applying the analytical framework	The working analytical framework was applied by indexing subsequent dyadic analyses (a word document consisting of partner and man with PCa summaries) using existing categories and codes. The analytical framework was created after six tables of dyadic analyses had been developed from the couple transcripts.
Stage 8: Interpreting the data	The variation in experiences of couples was explored based on specific factors of interest and using dyadic theory to guide interpretation and analysis, for example, treatment type, age group, and length of marriage. Other factors that were explored included highlighted concerns, similarities, and incongruences across couples (Eisikovits & Koren, 2010), and what is unique to younger couples’ experiences.

*Note.* PCa = prostate cancer.

The stages of the analysis process, which reflect the evolution of the framework development, are shown in the Supplementary File. Explanations are provided detailing each part of the process as listed in the stages above.

## Reflections on the Dyadic Analysis Process

In this section, we discuss our reflections on the dyadic analysis process we followed, suggest ways we could have better conducted the analytical process, and implications for the way future dyadic analyses could be conducted.

### Contributions of Dyadic Analysis

Conducting dyadic analysis using the Framework method yielded interesting results by highlighting the dynamics of relationship processes in couples. Stage 5 of the analysis process—“dyadic analysis,” in which dyadic summaries were created allowed for a clearer understanding of couples’ perceptions of their experiences at a dyadic and individual level, what coping mechanisms they put in place to manage their experiences together and what challenges they faced. From this analysis process (see Supplementary File Stage 8), an overarching theme was developed “evolving couple identity.” Couple identity refers to the sense of “us” or “we-ness” in the relationship. A further three key themes were developed: “Couple Relationships—Integrating/Managing Old and New Relational Dynamics”; “Work and Finances: Challenges, Buffers, and New Directions”; and “Development of Social Connections and Impact on Social Activities.” The impact of PCa on younger men and their partners led to significant changes to couples’ relationships, parenthood and family functioning, work and finances, social activities, and connections. These impacts triggered various engagement strategies and behaviors within couples’ relationships which influenced their adjustment to PCa, and therefore couples’ sense of “we-ness,” their shared identity as a couple. A third overarching theme was also developed from the findings relating to treatment and health care issues; however, for the purpose of the article (Collaço et al., 2020), the focus was on the overarching themes: “Evolving Couple Identity” and “Couple Engagement Strategies and Behaviors.”

Furthermore, Stage 8—interpreting the data using theoretical frameworks on couple adjustment guided the analysis and interpretation further through highlighting similarities, differences, and what was new across existing theory and the findings from our data regarding relationship processes and adjustment. For example, our data highlighted that couples employed specific engagement strategies and behaviors to adjust to the impact of PCa on their lives (e.g., relational communication, distancing from unfamiliarity, mind-set toward PCa, and distraction), sharing similar findings with Manne and Badr’s (2008) relationship intimacy model of couples’ psychosocial adaptation to cancer. Drawing upon such dyadic coping models enhanced our understanding of why couples may engage in such behaviors in the context of younger men and their partners affected by PCa, as well as providing insights such as how to better support these couples.

### Frameworks

The Framework method was useful and appropriate for the aims of this study. Placing information from the table of themes into the Excel spreadsheet which consisted of the framework matrices allowed for clear recognition of where codes could be combined, created, or deleted, and other patterns in the data identified. The initial process generated 11 frames, of which one was termed *other* for codes initially difficult to place. For example, initially, separate themes were created for “managing emotions from changes to intimate relationships” and also the “psycho-emotional impact” which included emotional impact on relational aspects of the couples’ experiences. After discussion with the co-investigators of this article, codes relating to managing emotions from changes to intimate relationships were placed under the “Relationship” theme. The systematic procedure of the Framework method makes the process easy to follow, especially with the large dataset for this qualitative study. Its flexible process means that reflexive notes can be considered more carefully within the matrix which added more depth and understanding of the phenomenon under study.

The analysis process of dyadic data was initially experimental, and we were therefore developing the process as the analysis continued. Initially, one-sentence summaries for the codes were created, but after further analysis and creation of codes, we realized that more information was needed in the dyadic codes/summaries, as the context was not always clear. As there were no quotes in the Excel spreadsheet of the framework matrices, the dyadic codes became less clear and lost contextual meaning; therefore, we went back to the original transcripts and ensured more detail was placed in the summary tables. Adding detailed quotes in the summary table provided more context and clarification of the summaries. Providing this level of detail at this stage reduced the need to look back at transcripts too often along the analysis process.

Precautionary steps were taken at each stage of the analysis. For example, care was taken in adding line numbers for each quote from the original transcript to the table of themes, highlighting nuances that made the process more time-consuming, such as color coding reference to gender norms, the impact of cancer on younger men and their partners, and codes that overlapped (see Supplementary File). On reflection, using a computer package such as NVivo to aid in the development of the framework matrices stage of the analytical process could have been helpful in addressing the time element of this process, particularly for a large study involving researchers from different institutions (Welsh, 2002). NVivo can be used to link the summaries to the relevant part of the transcript making it easier to work through the data. Initially, we used NVivo to code the data; however, when moving forward to create dyadic summaries, we found its use restrictive because the transcripts for both the man with PCa and his partner were carried out separately and we were therefore unable to bring the data together to create a table of themes using this software. Microsoft Word and Excel served well for managing data analysis for this study.

### Challenges in Conducting Dyadic Analysis

Bringing together the experiences of couples interviewed separately was a more complex process than anticipated. The first challenge was analyzing members of the couples’ different perceptions of one another’s experiences. For example, some members of the couple had different perceptions of communication within their relationship:

You know when the treatment started I had to push all the time, well how was it today?, how’s treatment?, but he didn’t share anything. He kept it all in and he really didn’t want to talk about it a lot . . . (Wife)

If I’ve got an issue or she’s got an issue, we can talk quite openly. If she’s got a concern about something, then we’ll bring it up and talk about it . . . (Husband)

This highlighted their differences in perceptions of relational communication. It could be that the husband or wife was presenting to the interviewer a certain view of themselves for fear of being judged, or that they have different perceptions of how they would define their level of openness with each other. A solution we developed to account for these differences in perception was to code these views under a general/broad term, for example, in this instance: “Relational communication.” When conducting Stage 8—interpreting the data of the analysis, the differing perceptions of certain parts of their experience were incorporated in the dyadic summary.

We adapted a stage of Yosha et al.’s (2011) analysis by creating a table with patient and partner quotes (Columns 1 and 2—see Supplementary File) for the initial stages of our dyadic analysis. A third column was also created to establish the dyadic code/summary of the couples’ experiences, so in that way we were able to bring individual accounts of the couple together. However, this approach presented difficulties in instances in which the researcher either had not asked the same question to both members of the couple to get a response from both that could be analyzed and therefore create a dyadic code/summary. Similarly, issues may simply have not been spoken about by the participant interviewed first, but was by their partner/wife (or vice versa), then information was missed out on when developing a dyadic code. It could be helpful to make notes on certain lines of inquiry in one interview that could then be followed up in the interview with their partner; however, confidentiality can become an issue (Ummel & Achille, 2016). A solution to address lack of uniformity in creating dyadic codes was identifying whether pertinent and related information had been recorded elsewhere in the dyadic table, and a dyadic code could then be created. If this was not possible, the key information was highlighted in another color under the most appropriate dyadic code and examined at a later stage upon further analysis of the data. Team discussions aided decision making about certain codes and themes. Keeping a reflexive journal promoted reflection on the interviewer role and how the data were analyzed in such a way as to reduce bias and maintain a level of objectivity (Berger, 2013).

Another challenge of this analysis was an initial overlap of codes throughout the different parts of the couples’ experiences. For example, one partner described difficulties in communication in relation to the process of treatment decision making. Therefore, this extract could be coded under “relational communication” and “treatment decision making.” It highlighted the challenges in separating experiences into simplistic categories. Experiences interconnect in many ways and are part of the whole experience of the couple. We realized our categories needed to be broader so they could be applied more clearly. To address this, the dyadic summary code names were kept broad and incorporated context of overlapping codes to provide further detail and depth to that part of the experience or impact.

Although the Framework method focuses on creating summaries, the process could potentially be rather descriptive. Creating dyadic summaries (Column 3—see Supplementary File) that differed from the subthemes column was difficult as there were some descriptions which could not be reflected in any other term but the subtheme code. For example, initially, we created a subtheme code called “Supporting wife.” The dyadic summary code was also termed similarly as it could not be described in any other way. Developing codes to a higher level of abstraction and moving forward from descriptive summaries to conceptualization was a challenging process. Theoretical dyadic literature (Badr et al., 2007; Manne & Badr, 2008) was used in the last stage of our analysis to move the data into a more conceptual interpretation; which also helped with grouping the data.

Ethical issues considered when conducting separate interviews and analyzing the data included the possibility that the couple might recognize his or her partner from the dyadic presentation of the data (Forbat & Henderson, 2003). However, we were careful to maintain confidentiality through anonymizing participant details through assigned ID numbers and removal of any identifiable details. Keeping partners’ versions confidential from each other prevented member checking from being conducted.

## Discussion

In this article, we have detailed and reflected upon the process of conducting a qualitative dyadic analysis using an adapted version of the Framework method, using our study on younger men diagnosed with PCa and their partners. We have demonstrated how examining individual narratives of both partners’ versions provides greater understanding of the variations and similarities between them. This allows for the creation of a dyadic version of their experience and a richer and more complete understanding of the couples’ relationships, their perspectives on shared experiences, the impact of their decisions and actions on each other, and the dynamics of their relationship.

Much of the literature that use the Framework method to analyze interview data when members of the dyad have been interviewed separately do not clearly identify how they bring together the individual transcript data to analyze the data at the level of the couple (Conroy et al., 2020; Patel & Agbenyega, 2013; Primeau et al., 2017; Swallow et al., 2011; White & Newman, 2016). Interestingly, some of the literature that uses the Framework method for analyzing qualitative data from members of a dyad who have been interviewed separately appear to incorporate additional stages to add to the analysis process (Starmann et al., 2017; White & Newman, 2016). For example, Starmann et al. (2017) created couple timeline maps of the sequence of relationship events for each couple from the transcript data. The map was used as a way to observe patterns and therefore identifies common themes and differences in relationship trajectories. However, it is not clear what such timelines would look like, how this could be replicated, and in what way the timeline could be used in a meaningful way to interpret the data. Furthermore, other studies (Starmann et al., 2017; White & Newman, 2016) have also used concepts from wider theory and literature to help understand key themes in the couple data—an approach we also incorporated in our analysis (Stage 8—interpreting the data). White and Newman (2016) also used Eisikovits and Koren’s (2010) process of dyadic analysis by comparing overlaps and contrasts within and between couples’ data, and the authors reflected on the benefit of this process in emphasizing the differences in couples’ relational styles. These articles highlight the lack of uniformity across studies in an approach to dyadic analysis, and how authors have in some way added in additional steps to the Framework method to analyze the data at the level of the couple, to gain a deeper understanding of the data.

As discussed in this article, challenges arose when conducting dyadic analysis with regard to maintaining confidentiality when bringing data together from both members of the dyad to analyze at the level of the couple and when questioning during interviews. Lack of clarity about how interpretations have been derived is a common criticism of qualitative research. However, we found that the Framework method, keeping a reflexive journal and sharing the reflexive process as a team allowed for a clear audit trail of the process. Reflectivity also allowed further insights into how best to explore the couple data at a more in-depth level and inform theme development and conceptualizations.

## Conclusion

The Framework method when utilized and implemented appropriately can be a suitable tool for conducting dyadic analysis and producing credible and relevant findings. New ideas from individual members of the dyad may direct us to interesting lines of inquiry or reveal variation in accounts of the nature of the dyadic relationship and the impact on their experience. A fluid and adaptable approach from all authors is essential for this form of qualitative dyadic analysis. There are methodological challenges, and the process is time-consuming and requires extensive reflexive and critical processing of participants’ thoughts and experiences in relation to existing dyadic theoretical concepts, and how these interpretations can be applied within the current condition of the health care system today. However, this type of analysis allows for a rich and deeper understanding into the complexities that exist in the nature of dyadic data, which can contribute to the improvement of health services and development of health policies.

## Supplemental Material

sj-pdf-1-qhr-10.1177_10497323211011599 – Supplemental material for Using the Framework Method for the Analysis of Qualitative Dyadic Data in Health Research Click here for additional data file. Supplemental material, sj-pdf-1-qhr-10.1177_10497323211011599 for Using the Framework Method for the Analysis of Qualitative Dyadic Data in Health Research by Nicole Collaço, Richard Wagland, Obrey Alexis, Anna Gavin, Adam Glaser and Eila K. Watson in Qualitative Health Research
