# Supplementary material for: Using the Framework Method for the Analysis of Qualitative Dyadic Data in Health Research
Source: Qual Health Res. 2021 May 13;31(8):1555–64. doi: 10.1177/10497323211011599 (PMC8278550; doi:10.1177/10497323211011599)
Supplement: sj-pdf-1-qhr-10.1177_10497323211011599 – Supplemental material for Using the Framework Method for the Analysis of Qualitative Dyadic Data in Health Research [file sj-pdf-1-qhr-10.1177_10497323211011599.pdf]

Supplementary File: Evolution of Coding Framework

1. Example Section of the Table of Themes Created for a Dyad

Column 1 ↓ **Column 2** ↓ **Column 3** ↓

	THEME			
	IMPACT ON RELATIONSHIP			
**DYAD**	**SUBTHEMES**	**Man with PCa**	**Partner/Wife**	**Dyadic code/summary**
15	***Support from wife***	Wife did the majority of information seeking related to PCa and treatment options (lines 93-94).	She attended every consultation with husband (line 142).	***Supporting husband***<br><br>She was husband's main source of support: she attended consultations with him and did the majority of information seeking in relation to treatment options. Wife reported having no one to turn to for emotional support. Family members would often offload their feelings onto her, and she therefore felt she had to support her family members by sorting it all out, pretending she was alright, and carrying on.
	Difficulties in balancing support		Felt unable to tell others when experiencing stressful situations. Everyone confides in her- she pretends to be okay and carries on: I *think that's the biggest problem for me is that you know, if there's something stressful going on, I can't tell anybody you know, everyone's going to tell me, including (husband), and everyone's going to dump it on me and I'm going to have to, just be Mum and rise above it all and you know sort it all out, pretend everything's alright and carry on and there have been moments in the last couple of years and that's been really hard* (lines 353-357).	
	Communication	He felt able to talk to wife about concerns or problems which he found helped him cope with his condition (lines 374-379).	She found difficulties in being able to discuss some of her worries with husband (secondary spread and possible outcomes) because he didn't/didn't want to know: *oh, the one person I usually talk to about everything that worries me is (husband), and I can't talk to him about worrying about secondary spread of cancer and what the possible outcomes of that could be because he doesn't know and he doesn't want to know, and I quite understand that, so that is difficult and was difficult* (lines 191-194).	***Dynamics of relational communication***<br><br>He felt able to talk to wife about concerns or problems which aided in his coping process. She would usually talk to husband about her worries, however experienced difficulties in discussing her concerns to husband about secondary spread and possible outcomes because husband didn't want to know/didn't know.
	Strained	He felt his relationship was strained in the	The relationship between him and wife got to a point	***Changes in the relationship***

After transcribing the interview audio files (stage 1), transcripts and reflective notes were re-read (stage 2). The third stage of the analysis consisted of separately coding the transcript of the man with PCa and his partner. Codes were either relevant to both members of the dyad or just one. The overall dyadic code/summary (see last column) encompassed a summary based on the individual codes from the man with PCa and his wife/partner. A general table of themes which included dyadic codes were created under each theme (framework) (stage 4). Initially themes created were based on the topics of discussion for example relationships, treatment etc. These were developed further after the initial stages of analysis were completed in order to be more conceptual.

2. Example Section of the Table of Themes for a Dyad with Reference to Gender Norms.

	THEME			
	IMPACT ON RELATIONSHIP			
DYAD	SUBTHEMES	Man with PCa	Partner/Wife	Dyadic code/summary
2	***Closer relationship***	The risk of dying from cancer brought the couple closer together: *'Umm it probably brought us a bit closer to be honest, ya know, because of the risk of death hanging over you'* (line 139-141).		***Relational communication*** Husband acknowledged that he found it difficult to communicate his feelings about his PCa diagnosis and attributed this to gender norms. Wife had not talked to husband about her worries because she side-lines her concerns and gets on with things. Wife reports that they did discuss PCa related topics and made decisions together, but not frequently- they are more likely to speak about PCa when nearing a PSA test follow up.<br><br>***Changes in relationship*** Despite husband's lack of communication he reported that PCa brought them closer together due to the possibility of death Wife felt there has been no negative impact on the relationship.
	No impact to relationship		Wife feels there has been no negative impact on their relationship (line 225).	
	Communication	*'[...] it were difficult because it were hard...it uh uh...were really difficult, umm just again, **just being a typical man we don't really talk, well I don't anyway, I don't really talk much.'*** **GENDER NORMS** (line 145-147).<br><br>Husband perceived no impact on the relationship from not communicating with wife about PCa: *[...] 'if I didn't talk about it, it didn't really put much of a strain, I don't think on the relationship because we're still together'* (lines 136-137).	Decisions were made as a couple through having discussions: *'I think, I think it was like it was happening to me you know, we discussed it together, we made our decisions together'* (lines 158-159).<br><br>Not talked to husband about worries *because I think, you know like at the back of my mind but you just get on with it, you just cope.* (lines 264-265).<br><br>Couple didn't talk about PCa frequently, only nearing the time of a PSA test follow up: *'[...] well we don't talk about it frequently but we might just mention it if it's getting near the time when he's having some tests.'* (lines 269-271).	
	Support from wife	Husband felt that his wife was supportive and coped well: *'I mean she's been great, she's there, I know she's there, we have a very close relationship anyway'* (line 201-202).	Regret over not being at the consultation when husband was diagnosed with PCa [they had young children who she was looking after at the time] (line 23).<br>She attended consultations with him: [...] *'and I always went with him to the biopsy's and the results of the biopsy's'* (line 201).	***Supporting husband*** Husband acknowledge the support provided by his wife. She attended follow up consultations with him. Wife reported regret over not being at the consultation when husband was

In instances where participants made reference to gender norms (see example), this was highlighted in another colour. This was considered further post-analyses and comparisons were made against other couples who also made reference to gender norms.

3. **Example Section of the Table of Themes for a Dyad with Reference to being Younger and its Impact.**

		chemo that killed him, if you know what I mean, it...they sorta got it but obviously...my aunty had *breast cancer and again it seemed to be more of chemo did it in more than...'* (lines 101-104).	cancer, you know the people that have done the best, *have been the ones' that have got it actually cut out rather than going down the chemotherapy route and things like that...* (lines 28-31).	
	Having young children influenced treatment decision process	Husband has young children which was an influencing factor in him wanting to have treatment rather than be on active surveillance: *'But it were just more of...I...we just had children, ya know, so it were more long term of me being around. It's alright blasting it with chemo now and coming back in ten years sort of thing.'* (lines 109-110).		
	Future concerns over cancer treatment: Age impact on body's ability to repair itself	View on treatment options in the future and the impact of age affecting these options: *'I know it's there, I'm expecting it at some point [...] in my life to have it removed, [...].* He feels that in 10 years he would like to have surgery because *[...] I think you get to sixty, stuff stops working, ya know stuff stops healing. I only got to look at me dad, ya know, your skin becomes like tissue (laughs), ya know what I mean, you get old, and I find. I don't want to wait till I'm old and can't recover from the surgery and my body be unable to repair itself* (lines 321-329).		
	Side effects		Initially couple reported being naïve in their understanding of the possible side effects of surgery:	

Reference to being a younger person and the impact of age on their experience of PCa was also highlighted. This was to keep focused on addressing the research question and objectives.

A table for 'OTHER' codes where the placement within the summary tables was not clear was created for each dyadic table, and returned too after all analysis was completed. It then became clear that some of the codes placed under OTHER could be collapsed into other codes under different themes, or in some cases a collection of these codes were created into a new theme.

4. Example Section of the Table of Themes for a Dyad which Highlights Overlapping Codes.

		noticed that I was literally just worn out (lines 553-558).	previously.
Communication	Husband felt able to speak more openly about his experience of cancer with a friend who had cancer than his wife because he found it difficult in talking about death with those he loves*: 'I can talk to my friends about it, he had cancer. I've got no fear of dying, I don't mind death, so many people have done it before me that I certainly haven't got to worry about it, I'd rather not go now but from a personal feeling um, my personal choice is that I'd rather not go today or tomorrow um but if that's what it was then that's what it is, um, it's very difficult to talk about things like that to be honest, to people you love* (lines 406-410).<br><br>His way of dealing with cancer was to ignore it which caused arguments and strained the relationship between him and his wife. There were times when he wanted to talk and she did not and vice versa, so he felt the best way to deal	Wife reported that husband withdrew from her. Initiating conversation with husband about PCa caused upset between them which she found difficult. In the period of discussing treatment options he dismissed surgery as he had concerns over being incontinent which she wanted to talk about and deal with his concerns together: *and if I tried to talk about it, he would just get angry, and he's not an angry man, he doesn't sort of shout and things, he just sort of withdraws and walks off, and that was so painful because it's really not what I wanted to do which I realise he was the one with cancer, you know, but um, I just felt like, I mean, you know, we talked about the options when the oncologist started talking about there was a possibility of surgery. I mean, he just said, I'm not doing, I don't want to be incontinent, that was it, end of story, but no, it's a bit more complicated than that, you know, and I'm following, running around behind him, sort of saying can we please talk about this* (lines 121-128). **OVERLAP TREATMENT DECISION MAKING.**	***Dynamics of relational communication***<br><br>When they found out about his diagnosis of PCa she was frightened and wanted to talk about it, however she felt she had no one to talk to about it with as husband became very distant towards her and did not want engage in conversation with her about it. In all other instances, they have pulled together and supported each other as a couple, however with the diagnosis of cancer he became detached towards her. There was a period of time where they had more discussions about how she was feeling, and she felt more able to show her upset in front of him rather than hiding these feelings.<br><br>His way of dealing with cancer was to ignore it. This coping process caused arguments and strained the relationship between him and his wife. There were times when he wanted to talk and she did not and vice versa, so he felt the best way

Some codes overlapped with others, therefore an acknowledgement of this was coded in red under the overlapping code (see screenshot). Dyadic summaries (last column in table) (stage 5) incorporated any overlapping codes to provide more context and depth to that part of the experience or impact. It is acknowledged that it is often not possible to separate experiences into simplified categories, as they are likely to overlap and interconnect in many ways, therefore overlaps in summaries exist so as to not lose the understanding of complexities that exist in experiences in general.

5. Example of Excel Framework Matrix for Theme Relating to 'Work & Finances'.

	Ability to work	Work adjustments	Reassessing the meaning of work	Financial impact	Conversations about PCa (colleagues/employers)
Dyad 2 Active urveillance	He is self-employed and felt that his work life was not affected as the PCa didn't affect him physically.	Couple discussed future implications to work: wife would work full time due to physical implications on husband from surgery.		Financial back up plans put in place should husband's ability to work be compromised- Couple saw a financial advisor in which critical illness policy was discussed.	
Dyad 19 RP	Experiencing erectile dysfunction and incontinence affected his self-esteem and self-confidence which in turn affected his ability to work. Subsequently, he took time off work (about a month).	Post surgery he was off from work for 3 weeks and then working part-time which suited him as he was frequently going to the toilet due to the side effect incontinence, which impacted on his work when visiting clients.	Their work life was impacted for the better as husband doesn't work the long hours he used too. Subsequently, this impacted on wife's life positively as it made them readdress their lifestyle and as a result they both do not work long hours.		
Dyad 10 RP, EBR	Whilst he felt that there was no impact to his work life, wife struggled to concentrate at work as she worried about his diagnosis of PCa and the possibility of not having children.			No financial implications from experience of PCa.	He was open about his diagnosis and PCa experience to colleagues and prompted colleagues to also get tested as they were a similar age to him. Colleagues were supportive of him and would also chat to him about PCa.

... | IMPROV SUGGESTIONS | FAMILY & FRIENDS | SOCIAL | DIET & LIFESTYLE | WORK & FINANCES | BENEFIT FINDING ...

Dyadic summaries were placed into framework matrices relevant to the themes. The frameworks were created using Excel, with tabs created to reflect the different themes. The initial framework matrices were: Relationships, Adjustment processes, Psycho-emotional impact, Healthcare service, Support & Information, Improvement suggestions, Family & Friends, Social, Diet & Lifestyle, Work & Finances, Benefit Finding and Other.

6. The Development of the Framework Called 'Family and Friends'

	Implications on children	Talking to others about PCa	Support from family and friends/community
Dyad 6 LP, EBR	Couple had a different perception as to how their children were impacted by the husband's diagnosis of PCa. Husband noticed that their children reacted in different ways, i.e. daughter was more defensive and son took a passive approach. Wife's perception was that their children were positive about things due to their grandfather's recovery from PCa.		Friends have been supportive in offering lifts to the hospital when needed, and checking up on couple after husband's treatment.
Dyad 7 EBR & HT			Family and friends provided emotional support to couple, by listening to them and offering help with practical activities such as gardening.
Dyad 8 RP		Husband felt that some people don't want to talk about cancer, and therefore did not bring it up. Wife did not feel able to confide in just anybody about her PCa experience as she felt that people who had not been through it themselves would not understand or be able to relate.	Wife reported a lack of support from friends as they seemed to downplay the seriousness of his condition which she found upsetting.

... IMPROV SUGGESTIONS | FAMILY & FRIENDS | SOCIAL | DIET & LIFESTYLE | WORK & FINANCES | BENEFIT FINDING ...

Dyadic code/summaries were placed under the appropriate subtheme in the specific framework.

Initially, there was code called 'implications on children' located under the framework: family and friends. As the analysis developed and further dyadic analyses was carried out, it seemed that the impact on the role of being a parent with cancer and on their children was a theme in itself, as there was detailed information on different aspects of this particular impact.

6.1 Theme and Subtheme Development for Matrix: Parenthood and Family Functioning

	Conversations about PCa	Impact on children	Challenges of being a parent affected by PCa	Hereditary implications	Desire for children in the future	Support
Dyad 1 Surgery, EBR, HT	Couple did not explain to children that father had cancer due to their young age, however were open with informing them when husband was going to the hospital.		Husband's motivation to keep going with treatment was that he wanted to see his daughters grow up, however this was an element that changed throughout his PCa experience in that he started enjoying being in the moment and enjoying time with his daughter's now rather than worrying about their future as a family.		Couple wanted to try IVF treatment because they had planned to have more children and didn't know what their possibilities would be once husband had rreatment.	
Dyad 5 Chemo & HT		Parents reported that their son struggled to come to terms with his father's diagnosis and negatively impacted his engagement and focus in school.	Husband withdrew from son because of how he was feeling about PCa- him being a younger man with PCa and the possibility of not seeing his child grow up. Subsequently, withdrawal from son caused conflict in the couple as they argued about the time spent together as a family.<br><br>Couple reported on the difficulties and stress of having a young child and dealing with a diagnosis of PCa at this point in their life.	Couple worries about the implications for son's future regarding PCa. Husband was told by a doctor that his son should think about getting a PSA test from the age of 40.		

... | TREATMENT | SUPPORT & INFO | IMPROV SUGGESTIONS | FAMILY | PARENTHOOD & FAMILY FUNCTIONING | SOC ...

In response to this, a further framework termed: Parenthood and Family Functioning was created to highlight this being a separate framework. Subthemes *Conversations about PCa, impact on children, challenges of being a parent affected by PCa, Hereditary implications, desire for children in the future and suppor*t were subthemes created under this theme.

6.2 Example of how Framework Matrix was Developed to Refine Subthemes and Subcodes within this Theme

Conversations about PCa

Sub themes	*Subcode*
Barriers to discussing PCa	○ *Perceived emotional impact*<br>○ *Child's life stage (being too young, at an age where they have a lot going on e.g. exams, university)*<br>○ *Uncertainties around prognosis/ tx plans*<br>○ *Prior experience of losing someone to cancer*
Motivators to discussing PCa	○ *Lessen further worry and impact to child*<br>○ *Platform to talk with son about their worries over PCa*<br>○ *Keeping children informed*<br>○ *Helping children cope (being open, reassurance)*
Timing of informing children	○ *Differences in parent's views of when to tell child*<br>○ *Knowing the direction of treatment and outcome*<br>○ *Being ready to talk about it as a parent*
Strategies for discussing PCa	○ *Other sources (media)*

Parental reactions/responses to PCa impact

Sub themes	*Subcode*
Reacting to the impact posed by PCa	○ *Withdrawal from children reaction*<br>○ *Keeping family together reaction*<br>○ *Being the rock response*<br>○ *Stress of young family and PCa diagnosis response*<br>○ *Enjoying moments with children now reaction response*<br>○ *Keeping going to see children grow reaction response*
Practical impact on family life posed by PCa	○ *Tx impact restricting time spent with children reaction*<br>○ *Childcare concerns affecting support for husband reaction*
Hereditary implications	○ *Genetic testing response*<br>○ *Parental worry over son's future reaction*<br>○ *Involvement in research with the hope to make a difference to son's future response*<br>○ *Early PSA testing for sons*

Parental perception of the impact on children

Sub themes	*Subcode*
Psycho-emotional impact	○ *Worry over losing a parent*<br>○ *Shock*<br>○ *Depression*<br>○ *Withdrawal*
Differences in parental awareness of impact	
Differences in impact on siblings	

Levels of support

Sub themes	*Subcode*
Support from parents to minimise impact on children/young adults	○ *Reassurance*<br>○ *Maintaining normality*<br>○ *Getting on with life*
External support for children/young adults	○ *Accessing support*<br>○ *Space for young adults outside a life of cancer*

After further analysis and discussions with co-investigators, the codes were developed further to highlight the key aspects of this particular theme. *Conversations about PCa, Parental perceptions of impact on children, parental reactions/responses to PCa* and *levels of support* were the codes redeveloped. See above for how the subthemes were derived.

6.3 Example Section of Coding Framework for 'Parenthood & Family Functioning'.

2. **PARENTHOOD & FAMILY FUNCTIONING**

   2.1 Difficult conversations about prostate cancer

   Includes any information on the process of telling children about diagnosis.

   2.2 Parental perceptions of impact of on children

   The parents perceived impact of being a child to someone affected by prostate cancer and the impact on their lives.

   2.3 Parental reactions and responses to prostate cancer

   Includes any reference to behavioural changes of parent(s) towards child(ren), and changes in approach to how they spent time with their child(ren).

   2.4 The future of our family

   Includes any reference to the implications for sons of people affected by prostate cancer and PSA testing, as well as challenges related to couples desire to have more children.

   2.5 Levels of Support

   Any reference to support offered by children to parents and vice versa or outside support for children. This includes ways in which children try to *escape* cancer, e.g. going on holiday, to another safe space etc.

3. **SOCIAL**

   3.1 Social engagements

   Any reference to how their social life was impacted or not, excluding

Themes were refined further by looking over each dyadic summary. Creating subcodes aided the development of themes (stage 6).

This is part of the coding framework. Having this coding framework allowed for the analytical framework to be tested on other dyads (table of themes consisting of dyadic codes/summaries) and therefore development of further codes as required (stage 7). Comments were made and checked with co-investigators against the framework matrices before finalising codes. Descriptions were created for each code, and with further analyses, descriptions were developed to reflect the extent to which the couple experiences were portrayed.

7. Development of Coding Framework to Create Conceptual Themes

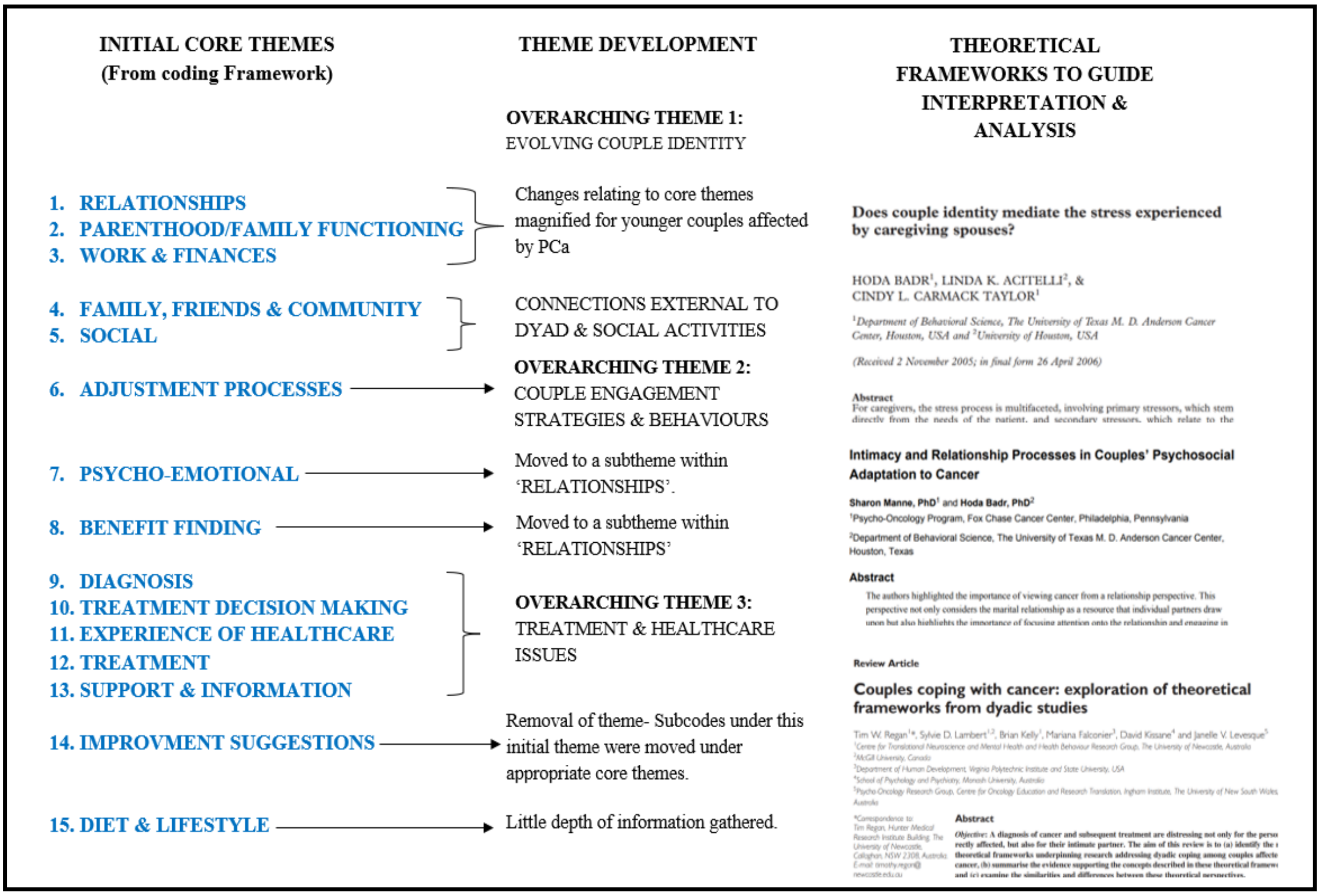

The initial themes in the coding framework aided in the development of three overarching themes, one relating to the impact of PCa and treatment on couples' lives, a second theme relating adjustment processes and a third about experiences of treatment and healthcare services. With the research question in mind, the first overarching theme was further developed to become "Evolving Couple Identity" including core themes that were unique/magnified to/in the experiences of younger couples affected by PCa (relationships, parenthood and family functioning, work and finances, social connections). The same process facilitated the development of core themes relating to the second overarching theme 'couple engagement strategies and behaviours' and third overarching theme 'treatment and healthcare issues.'

Themes and subthemes within these overarching themes were then further developed for writing as an article (Collaço et al 2020). The writing process highlighted where codes within these subthemes could be further collapsed. This, therefore, influenced changes to the structure, theme names and content of the themes presented for the article. Theoretical frameworks of adjustment and identifying overlaps and contrasts in dyadic narratives guided the analysis and interpretation further (stage 8).
